# Fellow-Workers and Their Doings

**Published:** 1914-02-21

**Authors:** 


### ROUND THE WORLD.

### Fellow-Workers and their Doings.

[The Editor Invites Early Information and Contributions Marked "Fellow-Workers."]

At Charing Cross Hospital Dr. David Forsyth continues to interest himself in the problem of hormones, particularly in regard to their effects upon growth and development. His investigations specially refer to the hormones of the ductless glands, to which he has for some time given special attention. Dr. Forsyth was, indeed, one of the first men to show clearly that the parathyroid glands are functionally independent of the thyroid. The regard with which his work is viewed in scientific circles is evidenced by the fact that he is a member of the London Medical Research Club, a small private association of leaders in active medical research.

Dr. A. D. Firth, M.B., M.R.C.P., who has succeeded Dr. Langmead as assistant physician at the Royal Free Hospital (London School of Medicine for Women), has had experience of several important appointments. These include the posts of out-patient physician to both the City of London Hospital for Diseases of the Chest and the Victoria Hospital for Sick Children. Dr. Firth pursued his medical studies at Cambridge and St. Thomas's Hospital, having subsequently served on the resident staff of the latter institution; he has also been a resident at the West London Hospital, where he has lately held the appointment of medical registrar.

Dr. E. F. Buzzard's services should be welcome to the Royal Hospital for Incurables at Putney Heath, to which he has just been appointed a consulting physician, for in such an institution there are always many sufferers from chronic nervous diseases, whom his experiences as an eminent neurologist particularly fit him to assist. Dr. Buzzard, who is a physician to out-patients at St. Thomas's Hospital, has for some years been on the staff of the National Hospital for the Paralysed and Epileptic, in Queen Square, where his father, Dr. Thomas Buzzard (now retired), did excellent work before him.

Dr. Nathan Raw, M.D., M.R.C.P., the well-known Liverpool physician, has lately been considering the problems of tuberculous rheumatism, an example of which he recently brought before the Royal Society of Medicine. Dr. Raw stated that in his experience he had only seen two other cases of this rare disease, one at Washington and the other at Berlin. The chief characteristic of this malady is a migratory inflammation, due to tuberculosis, affecting many joints, and commonly following primary infection of the cervical glands.

Dr. Gordon Ley, who has lately been appointed pathologist to the Chelsea Hospital for Women, is giving special attention to the important subject of the classification of ovarian cysts. This has, so far, been based mainly upon conjectures as to their embryological origin, but it is possible that a grouping founded on histological characteristics will be simpler, as well as more scientific. Dr. Ley, who was formerly resident medical officer at Queen Charlotte's Hospital, is one of the comparatively few practitioners who can write M.R.C.P. as well as F.R.C.S. after their names, is collecting records of the microscopical appearances of hundreds of specimens, and from his observations hopes to evolve a new and more useful grouping of ovarian tumours.

The work of one of our most foremost neurologists, Dr. F. W. Mott, M.D., F.R.S., has once more received material recognition abroad, as shown by his election to be a corresponding member of the Paris Neurological Society. It will be remembered that Dr. Mott, who has lately retired from active service at Charing Cross Hospital, has for many years directed the research laboratories of the London County Council at Claybury, where he and his fellow-workers have done much to advance our knowledge of neuro-pathology, and have, indeed, placed the physical conception of certain forms of insanity on a scientific basis. Fortunately for other investigators, the pioneer work of Dr. Mott and his assistants is permanently preserved in readily accessible form in the "Archives of Neurology," which have been published under his authority.

Dr. A. H. Gosse, M.B., M.R.C.P., who has just been succeeded in the medical registrarship at St. Mary's Hospital by Dr. H. G. B. Castellain, is now working in the cardiological department at the London Hospital under Dr. James McKenzie. Dr. Gosse was born in South Australia, and educated at St. Peter's College (Adelaide) before he went to Cambridge and St. Mary's.

Dr. F. F. Muecke is another Australian who has recently come to the front in London. He is an M.B., B.Ch., of Adelaide University and an F.R.C.S. Eng. Dr. Muecke has devoted special attention to throat, nose, and ear diseases, and now holds the appointments of assistant surgeon to the London Throat Hospital and aural demonstrator to the London Hospital. His present investigations are particularly interesting as he is on the committee which is inquiring into the question of the ventilation of the House of Commons. Dr. Muecke is the husband of Madame Ada Crossley, the famous contralto.

Dr. W. H. S. Armstrong, who is to be congratulated on obtaining the post of visiting superintendent to the Renfrew and Clydebank Joint Hospital for Infectious Diseases, is an M.D., Ch.B., of Glasgow, where he has held the appointments of house-physician and casualty surgeon to the Western Hospital.

In these days of complicated anæsthetic apparatus, and increasing tendency to dosimetric devices, it gives food for thought to note the comfort and safety with which some of our best known senior men—for example, Dr. Dudley Buxton, at University College Hospital—carry on their work with simple routine methods. And it is noteworthy that more than one exponent of the older school seems content with the simplest of drop-bottles, sometimes using nothing more than an ordinary phial, fitted with a cork in which two small lateral channels have been cut. There is doubtless not a little to be said in favour of the theory that in anæsthetics it is the man rather than the machine which is of chief importance.

The new honorary physician to the Royal Berkshire Hospital, Reading, Dr. G. S. Abram, has served that institution as assistant physician for some time. As a student of Cambridge and University College Hospital, London, Dr. Abram qualified M.R.C.S., L.R.C.P. in 1891, taking his double University degree (M.B., B.C.) in the same year.

